# Cultivable Fungal Endophytes in Roots, Rhizomes and Leaves of *Posidonia oceanica* (L.) Delile along the Coast of Sicily, Italy

**DOI:** 10.3390/plants11091139

**Published:** 2022-04-22

**Authors:** Livio Torta, Santella Burruano, Selene Giambra, Gaetano Conigliaro, Gaia Piazza, Giulia Mirabile, Maria Pirrotta, Roberta Calvo, Giancarlo Bellissimo, Sebastiano Calvo, Agostino Tomasello

**Affiliations:** 1 Department of Agricultural, Food and Forest Sciences, University of Palermo, 90128 Palermo, Italy; livio.torta@unipa.it (L.T.); santella.burruano@unipa.it (S.B.); selene.giambra@unipa.it (S.G.); gaetano.conigliaro@unipa.it (G.C.); 2 Institute of Life Sciences, Scuola Superiore Sant’Anna, 56127 Pisa, Italy; gaia.piazza@santannapisa.it; 3 Department of Earth and Sea Sciences, University of Palermo, 90128 Palermo, Italy; maria.pirrotta@unipa.it (M.P.); sebastiano.calvo@unipa.it (S.C.); agostino.tomasello@unipa.it (A.T.); 4 Italian Association for Water and Soil Bio—Engineering, Interregional Section Sicily—Calabria, 90139 Palermo, Italy; roberta.calvo@unipa.it; 5 Regional Agency for Environmental Protection of Sicily, 90149 Palermo, Italy; gbellissimo@arpa.sicilia.it

**Keywords:** marine meadows, dark septate endophytes (DSEs), *Lulwoana*, *Penicillium glabrum*, *Ochroconis*, *Xylariaceae*, Sicily

## Abstract

The presence of endophytic fungi in the roots, rhizomes, and leaves of *Posidonia oceanica* was evaluated in different localities of the Sicilian coast. Samples of roots, rhizomes, and leaves were submitted to isolation techniques, and the obtained fungal colonies were identified by morphological and molecular (rRNA sequencing) analysis. Fungal endophytes occurred mainly in roots and occasionally in rhizomes and leaves. *Lulwoana* sp. was the most frequent of the isolated taxa, suggesting a strong interaction with the host. In addition, eight other fungal taxa were isolated. In particular, fungi of the genus *Ochroconis* and family *Xylariaceae* were identified as endophytes in healthy plants at all sampling stations, whereas *Penicillium glabrum* was isolated at only one sampling station. Thus, several organs, especially roots of *Posidonia oceanica*, harbor endophytic fungi, potentially involved in supporting the living host as ascertained for terrestrial plants.

## 1. Introduction

*Posidonia oceanica* (Linnaeus) Delile is a Mediterranean paleoendemic in the infralittoral zone, where it forms extensive marine meadows from the surface to a depth of more than 50 m. [1], supporting highly diverse communities [2,3,4] and providing important ecosystem functions and services [5,6]. Most studies on seagrass-associated biodiversity have focused mainly on macro fauna and flora, revealing the high complexity of their communities, in accordance with the epi-hypogeal compartments of seagrass and the physicochemical gradient that they provide [7]. The microbioma associated with *P. oceanica* was studied from the end of the 20th century [8,9,10,11,12,13,14,15,16]. The results of these studies show that, like terrestrial plants, symbiotic bacteria and fungi colonize the host and that their populations vary according to organ, season, and environment.

The first studies of the presence of endophytic fungi in *P. oceanica* have highlighted the presence of the species of *Lulwoana* Kohlm et al. in the roots of plants of the central Mediterranean Sea [17], but none of the isolates have been identified as *L. uniseptata*. The new genus *Lulwoana*, which was previously included in the larger genera *Lulworthia*, typical marine ascomycetes include only one described species: *Lulwoana uniseptata* (Nakagiri) Kohlm et al. with the anamorph *Zalerion maritimum* (Linder) Anastasiou. *L. uniseptata* produces fusiform asci which are curved, deliquescing early and containing eight ascospores, which are filiform, hyaline, and one septate shorter than 150 µm [18]. In the root sections, inter- and intracellular melanized hyphae and microsclerotia, typical features of dark septate endophytes (DSEs) [19,20], were also observed. Subsequent studies have highlighted the presence of other endophytic fungi in the roots of the host and also detected *Lulwoana* sp. [21,22,23,24,25,26]. In most cases, the taxonomy, biology, and ecology of many of the isolated fungal taxa are not well-known. The detection of endophytic fungal populations in marine phanerogams (and in terrestrial ones too) could also help in understanding the type of symbiotic plant–fungus association (reciprocal, neutral, or antagonistic), considering the species of microorganism, the vegetative state of the colonized organ, and the environmental characteristics of the ecosystem. In particular, the fungal endophytes condition implies the absence of evident symptoms in the host; thus, it is important to assess the health state of the plants [27,28]. In this study the fungal endophytic symbiosis in *P. oceanica* was studied. Roots, rhizomes, and leave samples were collected from different marine meadows growing in three Sicilian seas, in order to (i) detect the presence of endophytic fungi also in these organs, (ii) confirm the presence of *Lulwoana* sp., and (iii) identify any other fungal taxa. The study of the endophyte community was designed in order to assess also the healthy vegetative state of the plant host by using leaf biometry analyses.

## 2. Results

### 2.1. Fungal Isolation and Identification

The agar plates used to test the efficacy of the surface sterilization technique showed no growth of epiphytic bacterial or fungal colonies, proving the adequacy of the employed method. The growth of fungal colonies from the vegetal fragments was detected up to 5 weeks after the assays were set up. From 7800 *P. oceanica* fragments, we obtained 504 fungi (absolute isolation frequency, IF = 6.41%). The total fungal population showed IFs (isolation frequency for the sites) of 12.3% for Bonagia, 78% for Ognina and 9.7% for Sciacca, while the IFo (isolation frequency for the organs) was 81% for roots, 17.9% for rhizomes, and only 0.4% for leaf samples (exclusively from Sciacca). Fungal isolates developing reproductive structures were morphologically identified at family, genus, or species level. Molecular analysis confirmed morphological characterization and was the basis for grouping fungal isolates into nine taxa (Table 1 and Table 2). 

Most of the fungal isolates (98.6%) were represented by 4 taxa: *Penicillium glabrum* (Wehmer) Westling (65.7%), *Lulwoana* sp. (23%), *Xylariaceae* Tul. & C. Tul (7.9%), *Ochroconis* de Hoog & Arx sp. (2%). All isolates of *P. glabrum* were obtained from plant roots taken from one of the two Ognina stations. Considering sampling sites and detected organs, the populations of *Lulwoana* sp., *Ochroconis* sp., *P. glabrum*, and *Xylariaceae* were distributed as reported in Table 3.

**Figure 1:**
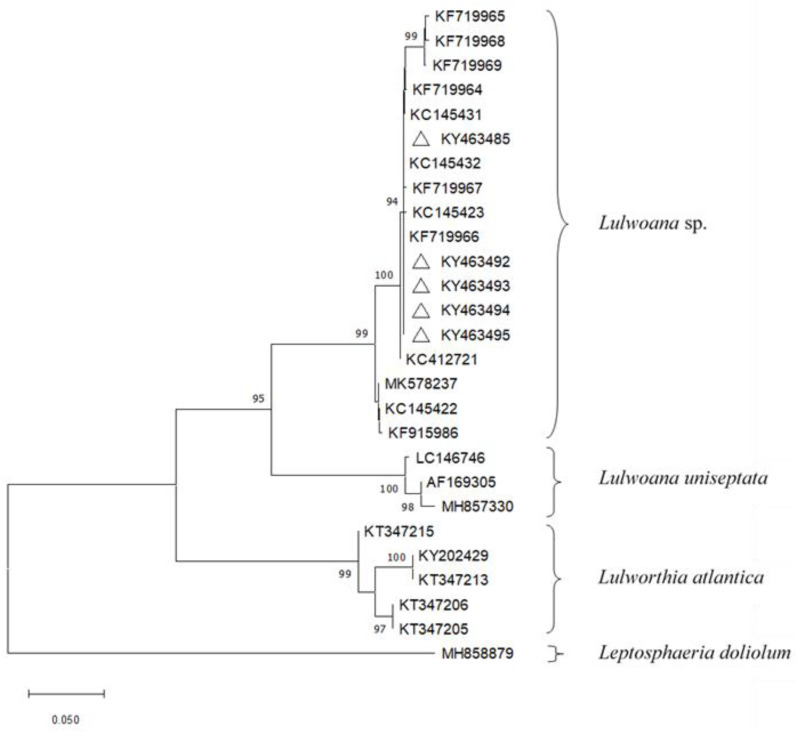
Neighbour-joining tree based on phylogenetic analysis of the ITS1-5.8S rDNA-ITS2 sequences of *Lulworthiales*. Bootstrap percentages calculated from 1000 re-samplings are indicated at nodes. GenBank numbers with triangles represent the sequences obtained in this study and deposited at the GenBank Database.

Phylogenetic analyses, based on ITS sequences exclusively, showed that the isolates of *Lulwoana* formed a separate subclade, with high bootstrap support, within a clade containing *Lulwoana* uniseptata Kohlm et al., (Table 4; Figure 1).

### 2.2. Detection of Fungal Endophytes in P. oceanica Organs

**Figure 2:**
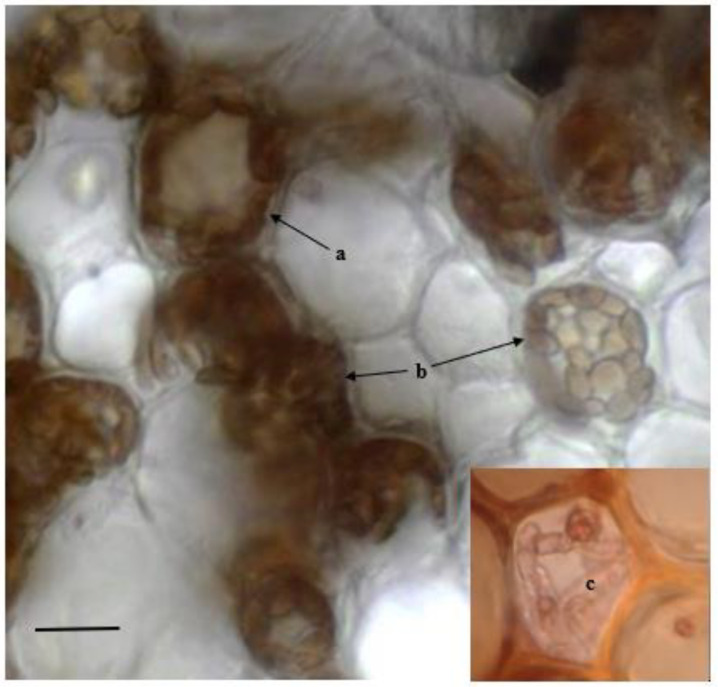
Radial section of *P. oceanica* root: intracellular fungal colonization by melanized and septate hyphae (a), brown microsclerotia (b) and young red-stained intracellular hyphae (c). Bar = 20 µm.

All the observed sections obtained from the sampled organs revealed the occurrence of several fungal structures, such as more or less melanized hyphae and microsclerotia, improved by the use of acid fuchsin. In particular, in almost all the observed root sections, melanized, septate, and thick straight hyphae on the surface produced extensive hyphal sheaths. Rhizodermal cells rarely contained single fungal hyphae, while most of the fungal colonization occurred inter- and intracellularly in the hypodermic and exodermal tissues, showing brown microsclerotia such as those described by [17] (Figure 2).

**Figure 3:**
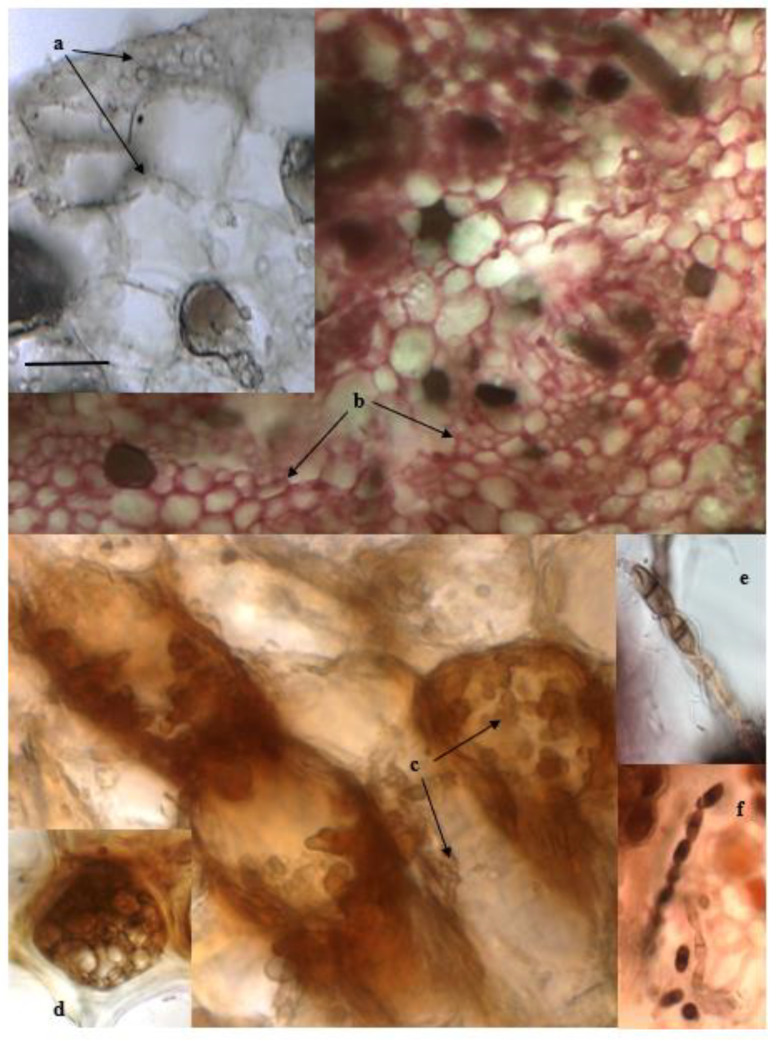
Section of rhizomes of *P. oceanica*. Melanized (a) and red-stained (b) hyphae surrounding and infecting exodermal and cortical cells, respectively. Detail of intracellular fungal infection by melanized and septate hyphae (c) and of a microsclerotium (d). Chains of melanized, two-cellular conidial structures outside (e) and inside (f) the rhizomal tissue. Bar: a = 50 µm; b = 100 µm; c, d = 20 µm; e, f = 10 µm.

Sections of the rhizomes from the three sites showed both melanized and grayish hyphae surrounding and enveloping the host cells, from the thin cuticle to the central stele. In the stained sections, the hyphae were coloured red. In a few cases, fungal propagative structures were observed, both outside and inside the detected organs (Figure 3).

**Figure 4:**
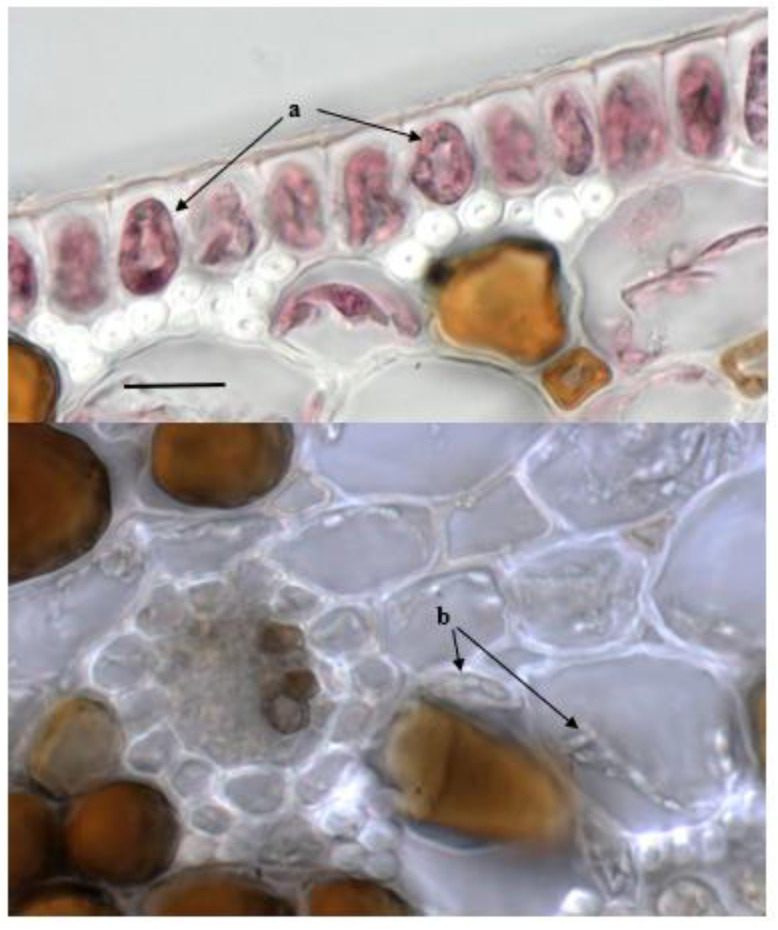
Section of leaves of *P. oceanica* from Sciacca: hyphae in epidermal (red-stained, (a)) and in mesophyll (not stained, (b)) cells. Bar = 20 µm.

Regarding fungal endophytic colonization in leaves, fungal structures were only detected in the sections taken from Sciacca samples. Thin hyphae infecting mesophyll tissue and epidermal cells (red-stained grayish compact microsclerotia in epidermal and, occasionally, in fibrous cells and hyaline-greyish hyphae in mesophyll tissues and in lacunar spaces were observed (Figure 4).

### 2.3. Leaf Biometry

**Figure 5:**
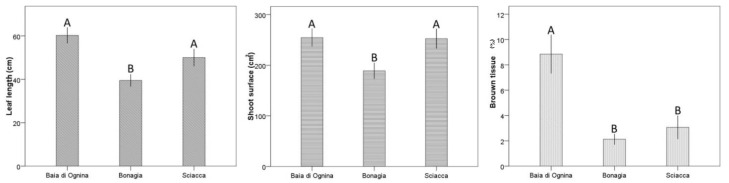
Mean (±SE) values of biometric variables at the sampling stations. Letters over the bars indicate a homogeneous group checked by a post hoc test.

The leaf biometry in the detected *P. oceanica* plants showed that leaf length varies from 39.5 ± 2.8 to 60.3 ± 3.7 cm and shoot surface from 189.0 ± 16.0 to 254.5 ± 17.7 cm^2^, while brown tissue is generally low, ranging from 2.1 ± 0.4 to 8.8 ± 1.5% (Figure 5). The low brown tissues are a normal feature in old leaves of *P. oceanica* in early summer. 

ANOVA detected statistical differences in the leaf length of shoots sampled from the different localities (*P* < 0.01), with higher mean values at Ognina and Sciacca than at Bonagia (*P* < 0.001; Table 5, Figure 5). Similar comparison results were detected for shoot surface (*P* < 0.05; Table 5), while brown tissue was bigger at Ognina compared to the other two localities (*P* < 0.05; Table 5). Biometric characteristics of leaf bundles showed on average values comparable to those recorded in a previous study [29] for the same three coastal sectors during the same season (current study: shoot surface 232 cm^2^ vs. 281 cm^2^ in [29]).

## 3. Discussion

Nine different fungal taxa were associated with seagrass, but only three of these recurred in the host as a result of isolation and identification tests. In particular, *Lulwoana* sp., *Ochroconis* sp., and some colonies ascribable to the *Xylariaceae* were differently distributed in the host population. Among these, only *Lulwoana* is known as a genus of obligate marine fungi [30], isolated also from submerged wood and driftwood [31,32].

The marine meadows chosen for this study are exposed to good or high environmental conditions according to a macroalgae-based index [33,34]. The slight reduction (less than 20%) compared to previous studies carried out in the same sector and period [29] is likely quite normal, considering that the study examined meadows growing mainly on sand and *matte*, which are known for 30% higher leaf length and shoot surface values compared to those growing on rock, as in our study [35,36,37]. Instead, observed morphometric variability between localities is a frequent phenomenon for *P. oceanica* meadows located hundreds of kilometres apart, reflecting differences in habitat type at the locations, such as wave exposure, temperature regime, and grazing pressure [37]. Moreover, among the three sampling areas, *P. oceanica* was more endophytized in the Ionian Sea (Ognina). 

The observation revealed that fungal colonization in plants is more abundant in roots, less in rhizomes, and only occasional in leaves. The study confirmed the presence of *Lulwoana* sp. in healthy *P. oceanica* roots in the Tyrrhenian Sea (Bonagia), as ascertained for the first time by [17], and in two other sectors: the Ionian Sea (Ognina) and the Sicilian Strait (Maragani). Moreover, the fungal endophyte is also associated with all the asymptomatic plant organs as well as the roots and rhizomes in all the tested meadows, and occasionally in the leaves (only in the Sciacca stand). *Ocrhoconis* sp. was associated with rhizomes (Bonagia and Ognina), roots (Bonagia), and leaves (Sciacca), whereas colonies of *Xylariaceae* were associated with roots (Bonagia and Ognina) and rhizomes (Bonagia). The distribution of these endophytes in the host is common to nonclavicipitaceous endophytes included in class II by [38] and, as such, could also play a fundamental role in maintaining the optimal vegetative state of the host. Moreover, the number of *Penicillium glabrum* colonies isolated from the roots of the plants in a spatial replicate of Bonagia could be due to special ecological conditions and require further study. The presence of all these fungal taxa as endophytes in *P. oceanica* is reported for the first time.

Although ITS sequences could not clearly discriminate all fungal taxa at species level, phylogenetic analyses showed that our strains of *Lulwoana* sp. isolated from *P. oceanica* belong to a similar cluster represented by *L. uniseptata*. Our *Lulwoana* sp. isolates clustered with other strains were isolated from *P. oceanica* in Italy, France, Croatia, Spain [17,22,39] and from driftwood in Italy [31]. Further analysis using more specific primers is clearly needed for better identification of the species to which our isolates belong. Moreover, considering the studies of [24,32], *Lulwoana* sp., like the other members of the *Lulwortiaceae*, produce cellulolytic enzymes, breaking down complex lignocellulosic compounds and thus contributing to the recycling of nutrients. 

Typical fungal structures of dark septate endophytes (DSEs) described by [20,30], such as inter- and intracellular melanized hyphae and microsclerotia, were detected in all the asymptomatic roots of the host, thus confirming their first detection [17]. Similar fungal structures were also detected in rhizomes. Other studies, subsequent to [17], also detected DSEs across the Mediterranean Sea in *P. oceanica*, including sites along the Sicilian coasts [26]. These results suggest that the occurrence of the fungal endophyte is more widespread than previously known.

The genus *Ochroconis* is characterized by melanized fungi producing rust-coloured to brown colonies, with brownish conidiophores bearing small numbers of septate, ellipsoidal conidia, mostly rough-walled, with sympodial conidiogenesis and rhexolytic liberation. All the species belonging to the genus are mesophilic and often oligotrophic, often reported from wet areas in the domestic environment or isolated from soil, water, humans, and animals such as insects and fish (*Salmo salar* L.) [40].

After recent taxonomic revisions [41,42], the *Xylariaceae* *sensu stricto* presently comprise 33 genera and over 1000 (and up to 1230) species, of which more than 50% belong to the genus *Xylaria* Hill ex Schrank. Most species display saprobic, pathogenic, or endophytic (in wood, leaves and fruits) behaviour or are associated with insect vectors, exhibiting their highest diversity in the tropics. The family *Xylariaceae* is also one of the most prolific lineages of secondary metabolite producers [43]. [44] described two new saprobic species of *Ascotricha* Berk belonging to *Xylariaceae* on the brown alga *Padina tetrastromatica* Hauck.

Although *P. glabrum* is a well-known pathogen and post-harvest agent of fruit and vegetable rots, frequently isolated from different matrices [45], some authors have also reported its presence in association with several hosts in marine environments. [46] detected it in soft coral, but it was also isolated from *Gracilaria lemaneiformis* (Bory) Greville (=*Gracilariopsis lemaneiformis* (Bory) Dawson, Acleto & Foldvik), *Sargassum thunbergii* (Mertens ex Roth) Kuntze [47], *Tethya aurantium* Pallas [48], and stems and rhizomes of the seagrass *Zostera marina* L. [49]. Regarding its ecological role, some studies have shown that *P. glabrum* can solubilize calcium and iron phosphates and have a xylanase activity [50], thus suggesting that it could play a role in the solubilizing and adsorption of these minerals, and in the decaying of dead plant tissue. 

For decades, studies of the presence and distribution of endophytic microorganisms and fungi in host plants, in particular, have contributed to understanding symbiotic relationships in nature. Their presence also appears to influence, among other things, the health of the plant they colonize. The importance of endophytic fungi in the evolution of plant communities was highlighted following the discovery of their relevant effects on the ecology, physiology, and development of each plant host. Above all, thorough research is needed to unravel the molecular mechanisms and signalling pathways undertaken by endophytic fungi to exert their effects on plants [27,28].

Also in marine environments, fungal communities could be considered as new frontiers in ecology, physiology, and chemistry studies. Fungal biodiversity has recently been detected in *P. oceanica.* This variability could be related to various factors (characteristics of the host, season, environment, etc.) and undoubtedly plays a well-defined role, which could involve the entire marine meadow. Here we show that *P. oceanica* organs represent a source of endophytic fungal biodiversity, prevalent in the roots and progressively decreasing towards the epigeal compartment, thus further corroborating the hypothesis of their involvement in substrate–host interaction [17,51]. It cannot be excluded that these results could also apply to other marine phanerogams. 

Therefore, it would be interesting to examine whether these fungal variability patterns are common trends within marine phanerogams. Further studies on this subject could provide information on the interaction between fungal endophytes and plants, which would be useful for gaining a better understanding of the functional implications at seagrass ecosystem level in healthy environments.

## 4. Materials and Methods

### 4.1. Study and Sampling Areas

**Figure 6:**
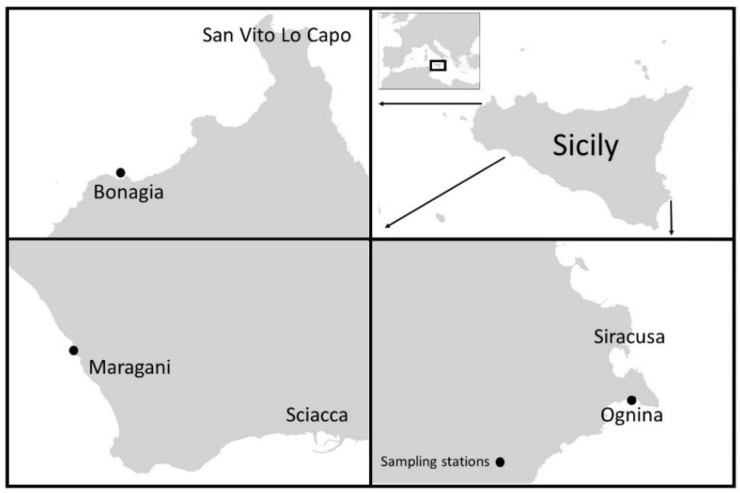
*P. oceanica* sampling locations along the coasts of Sicily.

In the summer of 2014, the study was carried out at three different localities along the coasts of Sicily (Italy), namely, Ognina on 14 July 2014(Siracusa, Ionian Sea: 526,863 E, 4,096,103 N 323,103 E, 4,155,293 N), Bonagia 21 July 2014(S. Vito Lo Capo, Tyrrhenian Sea: 287,646 E, 4,216,808 N), and Maragani 07 August 2014 (Sciacca, Strait of Sicily, Mediterranean Sea: 323,103 E, 4,155,293 N) (Figure 6). In these localities *P. oceanica* is exposed to very low human pressures because it is far from urban or industrial activities and from harbours or fish farms. Studies carried out in 2018 classified the water body of sampling stations on a scale ranging from “good” to “high” according to PREI and CARLIT indices, respectively [33,34]. At each locality, shoot samples were taken from meadows on a rock substrate at a depth of about 6 m at two stations located within an area of about 500 m^2^. Rocky substrates were chosen for sampling because increased fungal colonization has previously been observed on such substrates [17].

In particular, at each station, 5 asymptomatic *P. oceanica* shoots, with apparently healthy roots, rhizomes, and leaf bundles were randomly collected. The shoots were transported to the laboratory in tanks containing seawater and analysed within 24 h.

### 4.2. Isolation of Fungal Endophytes from P. oceanica Organs

After careful washing with tap water, *P. oceanica* roots, rhizomes, and leaf bundles were divided into two parts for use in isolation assays and cytological analyses, respectively. The first part was subdivided into fragments that were surface-sterilized by sequential washing in 5% NaOCl for 5 min, 95% EtOH for 1 min, and 5% H_2_O_2_ for 3 min, and then rinsed three times in distilled sterile water [52]. Prior to plating, a subset of samples was imprinted onto a fresh medium to check the surface-sterilization procedure. Previous investigations have shown that among the different artificial substrates Potato Dextrose Agar (PDA), Malt Extract Agar (MEA) [17], and Sea Water Agar (SWA, unpublished data), MEA was more suitable for the growth of fungal colonies. Therefore, sample fragments of 2 mm length were placed on 2% MEA (Oxoid, Milano, Italy) in Petri dishes (10 cm Ø, five fragments per dish). In total, for each plant the study analysed 75, 50, and 135 fragments of leaf, rhizome, and root, respectively [53]. The Petri dishes containing the fragments were incubated at 20 ± 1 °C in the dark, then observed on a daily basis for fungal development during the first 2 weeks and then weekly for 3 months. Subsequently, the dishes were checked occasionally, until the substrate dried. Hyphal tips from the developed fungal colonies were transferred to 2% MEA in order to obtain pure fungal colonies, morphologically identified macroscopically and microscopically using a light microscope (Axioskop Zeiss, Oberkochen, Germany) [54,55,56]. The isolation frequency of the fungal colonies (IF) was calculated using the formula: IF = (Nif/Ntf) × 100, where Nif is the number of colonies and Ntf is the total number of isolations attempted × 100. The isolation frequency for site(IFs) and the isolation frequency for organ (IFo) were also calculated. All the obtained fungal colonies were grouped by morphotype [53] according to their macroscopic and microscopic features.

### 4.3. Molecular Identification and Phylogenetic Analyses

One isolate for each morphotype was used for genomic DNA extraction applying the standard cetyltrimethylammoniumbromide (CTAB)-based protocol [57]. According to the standard method for fungal identification, the internal transcribed spacer (ITS) regions of the ribosomal DNA were amplified and sequenced with primers ITS1/ITS4 [58]. PCR amplification and sequencing of amplicons was carried out as described by [59]. Sequences were edited with Sequencher v 4.7 (Gene Codes Corporation, Ann Arbor, MI, USA) and compared with sequences deposited in GenBank through BLASTn searches. New sequences were deposited in GenBank (Table 1). Sequences of *Lulworthiales* currently known from cultures were retrieved from GenBank (Table 2) and aligned with sequences of the isolates obtained in this study. Phylogenetic analyses were performed as described by [60]. Alignments were made using ClustalX v. 1.83 [61] and, if necessary, manually edited using MEGA6 [62]. Maximum likelihood (ML) analyses were performed on a Neighbour-Joining starting tree automatically generated by MEGA6. Nearest-Neighbour-Interchange (NNI) was used as the heuristic method for tree inference, and 1,000 bootstrap replicates were performed.

### 4.4. Microscopic Observations

The second part of each organ was manually dissected in transverse sections (20 µm thick) and stored in an Eppendorf tube with a fixative solution (Formalin–Acetic acid–Alcohol, F.A.A.: 95% ethanol, 40% formaldehyde, glacial acetic acid, distilled water, respectively 30, 8, 2, and 60%). To visualize fungal structures, some sections were mounted in a lactophenol solution (25 mL distilled water, 25 mL glycerin, 25 mL lactic acid, 25 g phenol crystals) and directly observed. Other sections were acidified by rapid immersion in 10% (*v*/*v*) HCl in water, kept at room temperature in a lactophenol solution containing 1 gL^−1^ acid fuchsin for 10 min, washed with lactophenol solution, and mounted. Excess stain was removed by washing with lactophenol without dye [17]. All sections (20 for each organ and site) were observed using a light microscope (Axioskop; Zeiss, Oberkochen, Germany) coupled with an AxioCam MRc5 (Zeiss, Oberkochen, Germany) digital camera. Images were captured using the AxioVision 4.6 software package (Zeiss, Oberkochen, Germany).

### 4.5. Leaf Biometry of P. oceanica Samples

In order to obtain information on the status of the shoots under investigation, biometric analyses according to [63,64] were carried out on leaf bundles of *P. oceanica* sampled shoots. In detail, all leaves from each shoot were counted and characterized as ‘adult,’ ‘intermediate,’ or ‘juvenile’ according to the classes proposed by [63], and estimates of morphometric parameters were made following the procedures described by [65]. Measurements of leaf length and width and leaf necrosis were recorded. In this way, the mean shoot surface and the percentage of brown tissue with respect to the total leaf length were calculated.

### 4.6. Statistical Analysis

One way ANOVA [66] was used to detected differences in morphological variables. In particular, the factor of locality was fixed, with three levels: 1 Ognina, 2 Bonagia, 3 Sciacca. A post hoc mean comparison test (HSD Tukey) was performed, and significant differences were found (*P* < 0.05) by ANOVA. Prior to the analysis, the data were checked for homoscedasticity (Levene’s test) and transformed when necessary. The SPSS 14 package was used for statistical analysis.

## Figures and Tables

**Table 1:** List of fungal endophytes isolated from *Posidonia oceanica* meadows in three Sicilian seas with their corresponding GenBank accession numbers and Blast results, obtained from GenBank.

Isolate Code	Isolate Identity	GenBank Accession No.	Blast Match Sequence		
			Reference Accession No.	Coverage (%)	Identity (%)
PO1	*Cordycipitaceae*	KY463483	MH231248	99	96
PO2	*Fusarium* sp.	KY463484	MK589327	100	100
PO3	*Lulwoana* sp.	KY463485	KF719966	100	100
PO4	*Ochroconis* sp.	KY463486	MH063201	100	100
PO5	*Paecilomyces* sp.	KY463487	KF871460	100	99
PO6	*Penicillium glabrum*	KY463488	MH864674	100	100
PO7	*Sordariomycetes*	KY463489	GQ153240	100	100
PO8	*Thielavia microspora*	KY463490	JN709490	100	100
PO9	*Xylariaceae* sp.	KY463491	MK334345	100	100
PO10	*Lulwoana* sp.	KY463492	KF719966	100	100
PO11	*Lulwoana* sp.	KY463493	KF719966	100	100
PO12	*Lulwoana* sp.	KY463494	KF719966	100	100
PO13	*Lulwoana* sp.	KY463495	KF719966	100	100

**Table 2:** Composition of the fungal population (504 colonies) isolated from the different *P. oceanica* organs in the three study sites.

Taxa	No. of Isolates	IF%
*Penicillium glabrum*	331	65.7
*Lulwoana* sp.	116	23.0
*Xylariaceae*	40	7.9
*Ochroconis* sp.	10	2.0
*Cordycipitaceae*	3	0.6
*Fusarium* sp.	1	0.2
*Paecilomyces* sp.	1	0.2
*Sordariomycetes*	1	0.2
*Thielavia microspora*	1	0.2

**Table 3:** Distribution of *Lulwoana* sp., *Ochroconis* sp., *P. glabrum* and *Xylariaceae* population, as a function of sampling site and *P. oceanica* organ.

Taxa	No. of Isolates	IFo%			IFs%		
		Roots	Rhizome	Leaf	Bonagia	Ognina	Sciacca
*P. glabrum*	331	100	0	0	0	100	0
*Lulwoana* sp.	116	44.8	54.3	0.9	19.8	43.1	37.1
*Xylariaceae*	40	42.5	57.5	0	72.5	27.5	0
*Ochroconis* sp.	10	70	20	10	80	10	10

**Table 4:** Isolates included in this study. The newly generated sequences are indicated in bold font.

Isolate Number	Isolate Identity	Host	Country	ITS GenBank Acc. No.
RP2	*Lulwoana* sp.	*Posidonia oceanica*	Italy	KF719965
RP5	*Lulwoana* sp.	*Posidonia oceanica*	Italy	KF719968
RP6	*Lulwoana* sp.	*Posidonia oceanica*	Italy	KF719969
RP1	*Lulwoana* sp.	*Posidonia oceanica*	Italy	KF719964
P12	*Lulworthiales*	*Posidonia oceanica*	Spain	KC145431
P03	*Lulwoana* sp.	*Posidonia oceanica*	Italy	**KY463485**
P13	*Lulworthiales*	*Posidonia oceanica*	France	KC145432
RP4	*Lulwoana* sp.	*Posidonia oceanica*	Italy	KF719967
P03	*Lulworthiales*	*Posidonia oceanica*	Spain	KC145423
RP3	*Lulwoana* sp.	*Posidonia oceanica*	Italy	KF719966
P010	*Lulwoana* sp.	*Posidonia oceanica*	Italy	**KY463492**
P011	*Lulwoana* sp.	*Posidonia oceanica*	Italy	**KY463493**
PO12	*Lulwoana* sp.	*Posidonia oceanica*	Italy	**KY463494**
PO13	*Lulwoana* sp.	*Posidonia oceanica*	Italy	**KY463495**
P32	*Lulworthiales*	*Posidonia oceanica*	Croatia	KC412721
MUT 5413	*Lulwoana* sp.	*Posidonia oceanica*	Italy	MK578237
P02	*Lulworthiales*	*Posidonia oceanica*	Italy	KC145422
MUT 1483	*Lulwoana* sp.	Driftwood	Italy	KF915986
NBRC 32137	*Lulwoana uniseptata*	Submerged wood	Japan	LC146746
ATCC62580	*Zalerion maritimum*	Driftwood	U.S.A.	AF169305
CBS 280.54	*Lulwoana uniseptata*	Unknown	Unknown	MH857330
FCUL210208SF10	*Lulworthia atlantica*	Sea water	Portugal	KT347215
CBS 139632	*Lulworthia atlantica*	*Fagus sylvatica*	Portugal	KY202429
FCUL090707CF10	*Lulworthia atlantica*	Sea water	Portugal	KT347213
FCUL061107CP4	*Lulworthia atlantica*	Sea water	Portugal	KT347206
FCUL210208SP4	*Lulworthia atlantica*	Sea water	Portugal	KT347205
CBS 541.66	*Leptosphaeria doliolum*	*Rudbeckia* sp.	Netherlands	MH858879

**Table 5:** ANOVA results on leaf length, shoot surface, and brown tissue.

	Leaf Length			Shoot Surface			Brown Tissue		
Source of Variation	Df	MS	F	Df	MS	F	Df	MS	F
Locality	2	1863.9	9.5 ***	2	24,980.1	4.8 *	2	6.1	4.9 *
RES	47	197.1		47	5155.8		33	1.3	
Levene’s test	ns			Ns			*		
Transformation	None						Ln		
Post hoc test									
(1) Ognina (2) Bonagia (3) Sciacca	1 = 3 > 2			1 = 3 > 2			1 > 2 = 3		

RES = residual, DF = degrees of freedom, MS = mean square, F = ratio, *** = *P* < 0.001, * = *P* < 0.05, ns = *P* > 0.05.
